# Corrosion of Alumina-Spinel Refractory by Secondary Metallurgical Slag Using Coating Corrosion Test

**DOI:** 10.3390/ma15103425

**Published:** 2022-05-10

**Authors:** Sina Darban, Camille Reynaert, Maciej Ludwig, Ryszard Prorok, Ilona Jastrzębska, Jacek Szczerba

**Affiliations:** 1 Department of Ceramics and Refractories, Faculty of Materials Science and Ceramics, AGH University of Science and Technology, al. A. Mickiewicza 30, 30-059 Krakow, Poland; camille.reynaert@vesuvius.com (C.R.); ludwig@agh.edu.pl (M.L.); rprorok@agh.edu.pl (R.P.); ijastrz@agh.edu.pl (I.J.); jszczerb@agh.edu.pl (J.S.); 2 Laboratoire Matériaux et Durabilité des Constructions, INSA/UPS Génie Civil, Université de Toulouse, CEDEX 04, 31077 Toulouse, France

**Keywords:** corrosion, alumina-spinel, refractories, metallurgical slag, XRD, SEM/EDS

## Abstract

In this paper, the corrosion mechanism of commercial alumina-spinel refractory was investigated at 1350 and 1450 °C. Disc samples were coated with shells of two different slags containing 4 and 10 wt.% SiO_2_. The after-corrosion refractory was investigated in view of changes in its microstructure and phase composition by SEM/EDS and XRD techniques, respectively. At 1350 °C slags slightly infiltrated the microstructure, whereas at 1450 °C slags infiltrated the alumina-spinel refractory causing its significant corrosion. As a result of corrosion, new phases were formed, including calcium dialuminate (Ca_2_Al_4_O_7_), calcium hexaluminate (CaAl_12_O_19_), and gehlenite (Ca_2_AlSi_2_O_7_). Formation of calcium aluminate layers in the microstructure of the refractory inhibited further dissolution of alumina aggregates; however, expansive behavior of CaAl_12_O_19_ raised the microstructure porosity. The additional SiO_2_ in the slag doubled the amount of low melting gehlenite in the matrix, accelerating the corrosion process of alumina-spinel brick at high temperatures.

## 1. Introduction

The primary consumption of refractory materials—around 70%—is attributed to the steel industry. These high-temperature materials should have excellent chemical inertness, high thermal shock resistance, and adequate mechanical properties to show acceptable performance in aggressive and high-temperature environments in the steel industry, especially in a steel ladle.

Alumina-spinel bricks are refractory materials used in the sidewalls and bottom of steel ladles in secondary steel production. These materials directly contact the aggressive slag during the steel ladle operation. Previous studies have revealed that spinel is advantageous for corrosion resistance, and it prevents the dissolution of sidewalls of refractory materials in the steel ladle [1,2,3,4,5,6]. 

**Figure 1:**
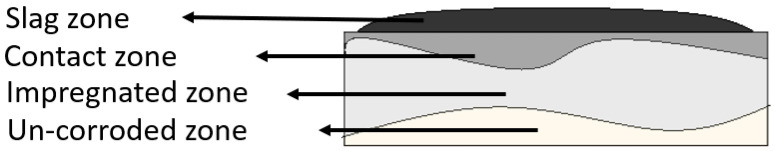
The schema of different corrosion zones in a contact corrosion test.

Chemical reactions and thermal gradients are two phenomena that take place—simultaneously—during work, and severely influence the corrosion process. Several zones can be distinguished within the refractory microstructure after the corrosion, including slag zone, reaction zone, impregnated zone, and unaffected zone, as shown in Figure 1. The thermal gradient affects the liquid viscosity, and it results in the crystallization of different phases in various temperature zones of the refractory. In most cases, the first stage in the corrosion of the refractory by liquid slag is its infiltration to the refractory through open pores. The open pores in the original refractory material facilitate infiltration of the liquid medium under the influence of capillary forces, and ease the diffusion process due to the larger surface area exposed to corrosion reactions. This infiltration often proceeds with the dissolution of grains starting from grain boundaries. The composition of the liquid phase, located mostly along the grain boundaries, is altered during the impregnation process. 

Refractories are designed to maintain their original texture and chemical composition. However, due to liquid slag infiltrating the refractory, both chemical composition and texture can be changed. Figure 1 shows zones that typically occur in corroded materials. The impregnated zone extends between the un-corroded and the corroded side where the dissolution of refractory components is followed by precipitation of secondary phases. 

The impregnation of a refractory by liquid slag can be limited by reduction of pore size in the refractory, or increasing the slag viscosity. However, applying the specific solution to preserve the refractory lining against infiltration must respect factors required by the metallurgical process [3,7,8,9].

Lee and Zhang [10] studied the corrosion mechanism of oxide and oxide-carbon refractories through static corrosion methods, i.e., sessile drop, dipping, and crucible test, as well as dynamic techniques, like the rotating finger or slag rotary test [7]. Their studies showed that two main mechanisms govern the corrosion of refractory materials. The first one is direct dissolution, in which the solid-phase from the refractory dissolves into a liquid slag with no intermediate phases, while the second is the indirect dissolution mechanism, in which new solid phases are excluded from the aggressive slag, which derives lesser risk of refractory spalling. Song et al. [11] studied the corrosion behavior of alumina-based refractories by sessile drop test in contact with SiO_2_-MgO-FeO-CaO-Al_2_O_3_-based slag obtained from smelting nickel laterite in a rotary heart furnace (RHF). They concluded corrosion in the system by three stages: melting and wetting, dissolution and diffusion, and final crystallization. The 0.43 and 0.63 basicity slags showed low apparent contact angles of 25° and 30°, respectively. They concluded that—for both types of slags—with extended corrosion time, two different covering layers formed between the alumina substrate and slag, preventing the further dissolution of Al_2_O_3_. The first layer was created by diffusion of Mg^2+^ and Si^4+^ from the slag towards Al_2_O_3_, while the second was formed by dissolution of Al_2_O_3_ and subsequent diffusion ofAl^3+^ into the slag. 

Braulio et al. [12] proposed a corrosion mechanism of spinel-containing castable in contact with slag of basicity 0.36. The pore size distribution was indicated as a crucial factor responsible for penetration of the refractory by slag and it was found that fumes of CaO and SiO_2_ reduce crack resistance by increasing slag infiltration. Martinez et al. [13] suggested that the formation of CA_6_ layers at alumina grain boundaries could potentially be helpful for improving corrosion resistance by inhibiting the refractory infiltration on the layer. From their perspective, the formation of CA_6_ and CA_2_ can be optimized by controlling the quantity of SiO_2_ fumes and using a proper binder, other than calcium aluminate cement. 

Corrosion of refractory depends on several factors, such as the composition as well as the viscosity of the slag, and work temperature, specifically, thermal gradient. So far, the majority of studies have concerned the slag corrosion at the working temperature of a steel ladle (1550–1650 °C); however, the thermal gradient in refractory is a determinant factor in the corrosion mechanism. The present study investigates the corrosion phenomena in alumina-spinel brick used in steel ladles at 1350 and 1450 °C; i.e., temperatures that actually occur at a cross-section of refractory during work. The alumina-spinel refractory was corroded by metallurgical slags, with one of them having increased acidity.

## 2. Materials and Methods

### 2.1. Preparation of Slag

Two slags of different compositions were used in this work, as presented in Table 1. The first slag was a steel ladle slag, delivered by the industrial partner in project ETN-ITN-ATHOR, designated as “R” slag. The second slag was prepared based on the composition of R slag by adding additional SiO_2_ (Sigma Aldrich 797863-5MG, St. Louis, MO, USA) to obtain 10% SiO_2_ in the slag—the slag is designated as “S” slag. The chemical compositions of R and S slags were measured by a Malvern PANalytical WDXRF Axios mAX spectrometer, Malvern, UK. A sample for XRF analysis was prepared by grinding a 5 kg batch of each slag, followed by its homogenization for 5 h. The main components of R slag were CaO (49%) and Al_2_O_3_ (41%), while SiO_2_ constituted 4%. The main components of S slag were CaO (46%) and Al_2_O_3_ (38%), while SiO_2_ constituted 10%. Thus, the amount of SiO_2_ in S slag was about 6% higher than in R slag. 

### 2.2. Alumina-Spinel Brick

Table 2 presents the chemical composition and physicochemical properties of test commercial alumina-spinel refractory. The main component of the test brick was alumina, which reached about 88%, the contents of MgO and SiO_2_ were at a similar level of 5%, while other impurities constituted about 2%. The refractory was characterized by 18.4% open porosity and 3.1 g/cm^3^ apparent density.

### 2.3. Corrosion Test

**Figure 2:**
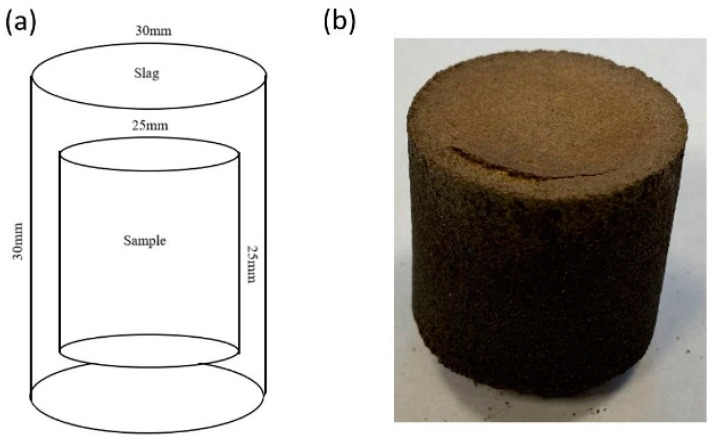
The samples for corrosion testing by the slag-coating method: (**a**) scheme of the corrosion system, (**b**) the commercial refractory sample covered with the shell of slag.

**Figure 3:**
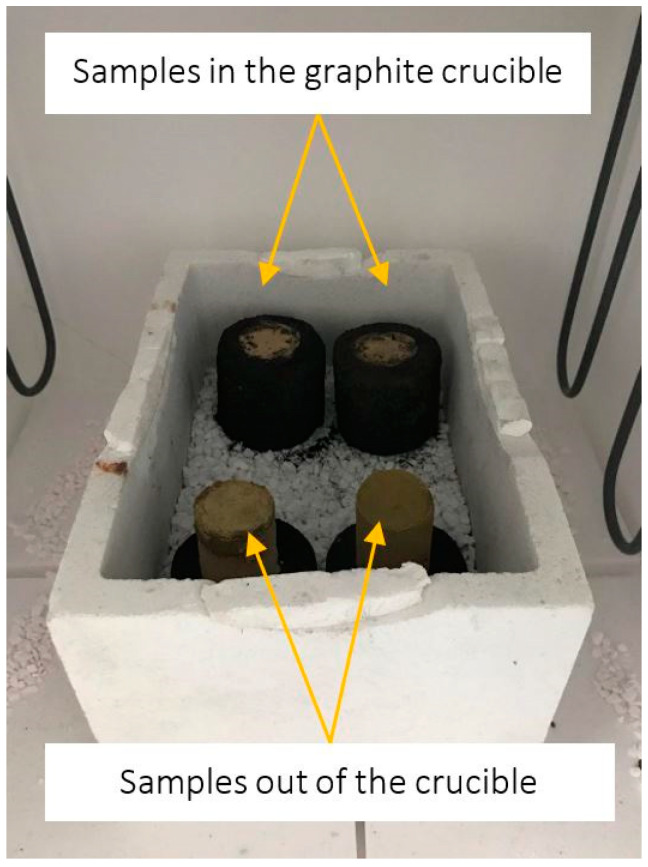
Samples prepared for corrosion testing, presented in two views: in graphite crucibles and out of graphite crucibles.

Figure 2 shows the schema of sample preparation for corrosion testing (left side) and the investigated refractory sample prepared accordingly (right side). Firstly, the alumina-spinel bricks were cut and drilled into disk-shaped samples (d = 25 mm, h = 25 mm) and dried for 24 h at 150 °C. Then, 20 g of each finely powdered slag (<63 µm; R and S) was pressed as a shell around the alumina-spinel disk samples, using a hydraulic pressing machine with a pressure of 50 kN. This methodology for corrosion testing was proposed by the author to maximize the contact between the slag and refractory sample, thus enhancing the impact of increased SiO_2_ content in the slag to the refractory. Another benefit of the applied method was ensuring that the liquid slag directly contacted the refractory at high temperatures. The prepared samples were heated in graphite crucibles (h = 55 mm, d = 35 mm) as shown in Figure 3. The slag did not react with carbon from the crucible because of the low wetting angle between slag and carbon. The corrosion tests were conducted in an air atmosphere, in an electrical furnace at 1350 °C and 1450 °C for 10 h with a heating rate of 5 °C/min. Table 3 presents the heat treatment conditions and designations of the corroded samples. 

### 2.4. Analysis of Material before and after Corrosion

The phase compositions of starting alumina-spinel refractory, slags, and after-corrosion materials were identified on powder samples using X-ray powder diffractometry (XRD). X-ray patterns were collected with an automated PanAnalytical X-Pert diffractometer (Almerow, The Netherlands) with Cu K_α_ radiation (λ = 1.5418 Å), in the 2θ range from 5° to 90°, in steps of 0.008°. The obtained diffractograms were analysed and matched with a database produced by the International Centre for Diffraction Data-ICDD PDF2, using X-Pert High Score Plus software v.3.0.5 produced by PANalytical B.V (Almelo, The Netherlands). 

The microstructures of the refractory, before and after corrosion, were investigated by scanning electron microscopy NOVA NANO SEM 200 (FEI, Hillsboro, OR, USA), equipped with an EDS system (EDAX). The samples for microstructure analysis were prepared by a standard ceramographic technique; resin-embedded specimens were polished and coated with a carbon layer. For delivered alumina-spinel brick, both polished cross-section and fractured section were observed. 

Hot-stage microscopy was employed to investigate in situ dimensional changes of slag R and S as a function of temperature. For this purpose, the homogenized powder slag was shaped into a cube of 3 mm dimensions. The measurement was conducted using a Carl Zeiss MH01 microscope (München, Germany) up to 1450 °C with a heating rate of 10 °C/min. The dimensions of the samples (height, width) were collected every 10 °C during the experiment and were used to prepare curves of in situ changes in cross-section dimensions of the test sample. The relative change in sample height ($\delta_{h}$) was calculated according to Equation (1). The sintering temperature was established as a dimensional change corresponding to 2% shrinkage of the sample compared to its original dimensions at 25 °C. The melting and flow temperatures were referenced to 2/3 and 1/3 of initial sample dimensions, respectively.

(1) $$\delta_{h}\left(T\right)=\frac{h_{\left(T\right)}-h_{0}}{h_{0}}\times 100$$

$h_{0}$—the initial height of the sample;
$h_{\left(T\right)}$—the height of the sample at a specific temperature.

## 3. Results

### 3.1. Reference Slag 

**Figure 4:**
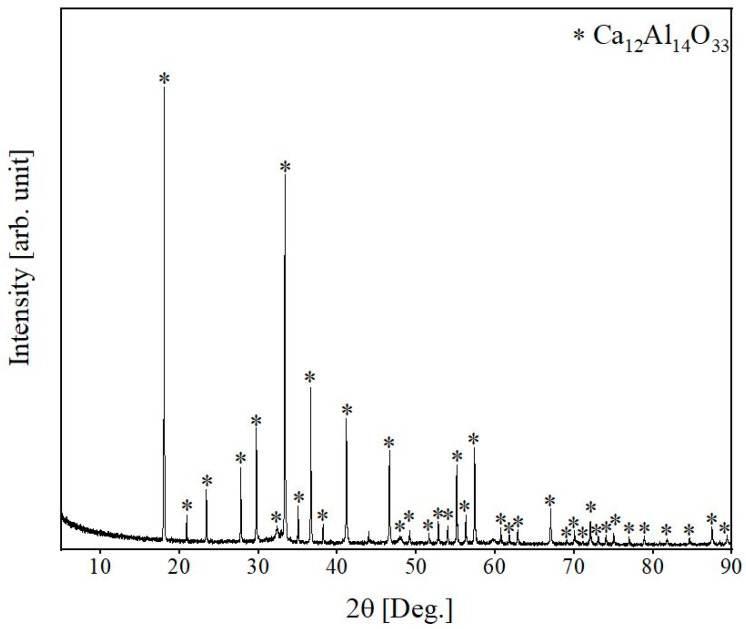
XRD pattern of R slag.

According to the XRD analysis of R slag (Figure 4), mayenite (Ca_12_Al_14_O_33_, denoted as C_12_A_7_), was identified as the only phase in the commercial slag. 

**Figure 5:**
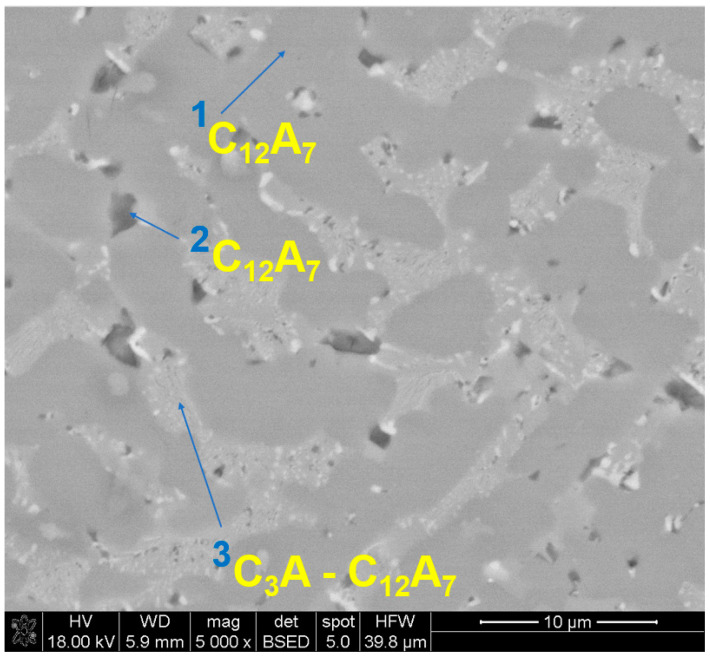
SEM micrograph with EDS in selected micro-areas for reference slag R.

In this study various calcium aluminates formed. The diagram CaO-Al_2_O_3_ consists of five binary phases: C_3_A, C_12_A_7_ (mayenite), CA, CA_2_ (grossite), and CA_6_ (hibonite), where C is CaO and A is Al_2_O_3_. Therefore, the stoichiometric aluminates can be characterized by different Ca/Al ratios, which are C_3_A-1.5, C_12_A_7_-0.9, CA-0.5, CA_2_-0.3, and CA_6_-0.1. Figure 5 shows the SEM micrograph of R slag. In the SEM images, three types of micro-areas of different contrast can be distinguished. According to the EDS chemical analysis in points 1 and 2, Ca/Al has the same value of 0.6, despite different contrast. They represent the mayenite phase, C_12_A_7_, which was also identified as the main phase by XRD; however, this phase is non-stoichiometric (Ca/Al_theor,_C_12_A_7_ = 0.9; point 1: 0.6, Table 4). The phase surrounding the mayenite is non-homogenous and contains a great number of small light inclusions. The atomic ratio in this area of Ca/Al equals 1.1, which indicates that it constitutes the non-stoichiometric phase from the system C_3_A-C_12_A_7_, with increased content of SiO_2_. 

### 3.2. Hot-Stage Microscopy Analysis

**Figure 6:**
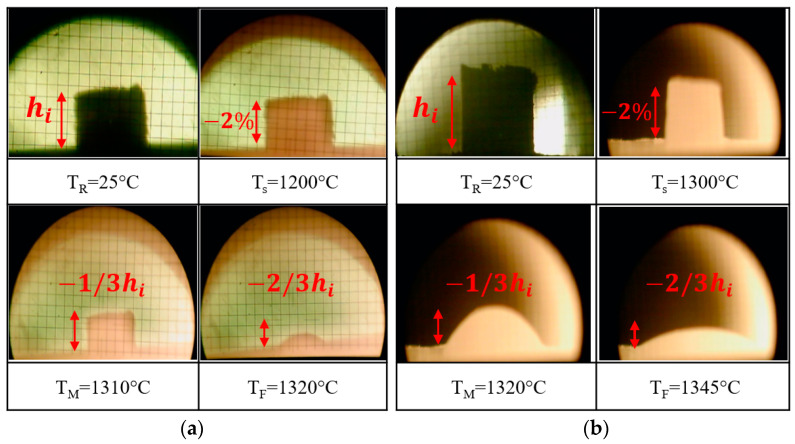
Hot-stage microscopy images of (**a**) R slag, and (**b**) S slag at selected temperatures (−2% h_i_—sintering, −1/3 h_i_—melting point, −2/3 h_i_—flow point).

Hot-stage microscopy is a widely implemented method in many research studies to identify the in situ high-temperature geometrical response of refractory material to slag, such as expansion or shrinkage during heating [14]. The heating microscopy permits the identification of sintering, melting, and flow temperatures to design the heating conditions of static corrosion testing. Figure 6a,b show the sintering (T_s_), melting (T_M_), and flow (T_F_) temperatures of R and S slags during the hot-stage microscopy test. 

Based on the obtained images, the R slag flowed around 1320 °C, while S slag flowed at a temperature 25 °C higher than R slag. According to these results, it was determined that the selected temperatures for the corrosion testing must be at least 1320 °C and 1345 °C for R and S slag, respectively.

### 3.3. Alumina-Spinel Refractory

**Figure 7:**
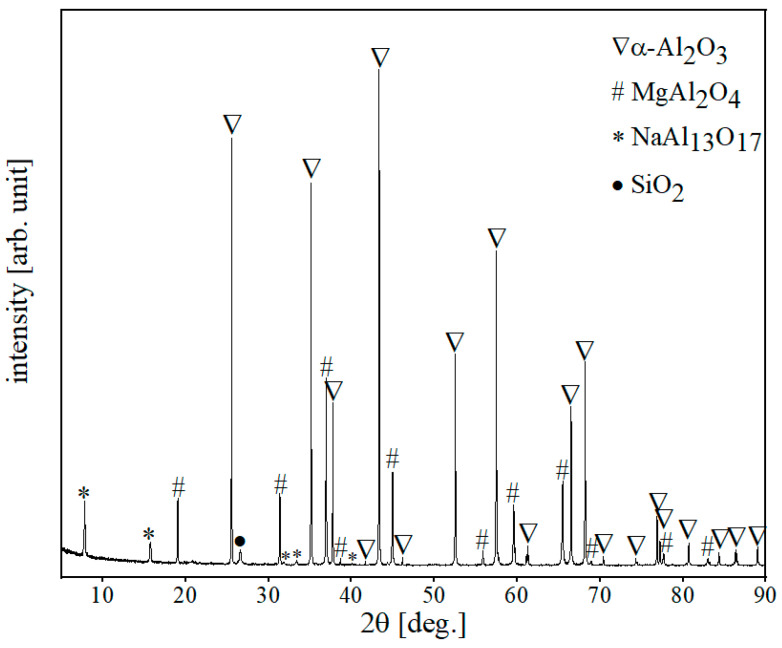
XRD pattern of alumina-spinel refractory.

According to the XRD results shown in Figure 7, corundum (αAl_2_O_3_) and non-stoichiometric spinel (Mg_7.5_Al_16_O_32_) were the main phases, along with beta-alumina (NaAl_13_O_17_) and silica (SiO_2_) as minor phases in the alumina-spinel brick. According to Jing et al. [15], the difference between spinel and alumina-rich spinel in the XRD pattern is a slight shift in the reflex position between 44° and 45° of 2θ. In our work, the reflex characteristic for spinel was shifted towards higher 2θ angles and equaled 44.95°, which confirms the presence of alumina-rich spinel in the test refractory.

**Figure 8:**
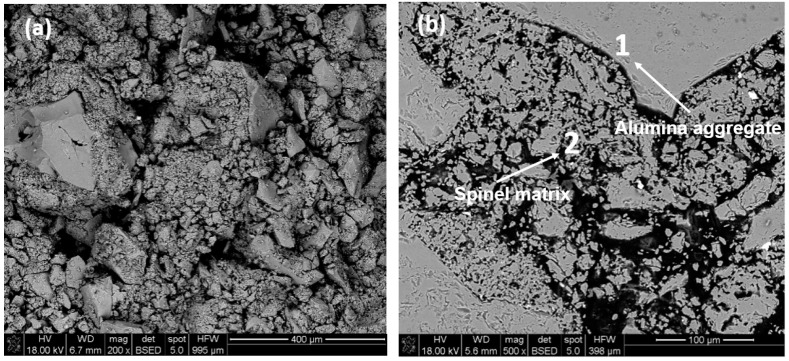
SEM micrographs with EDS analysis in selected areas of alumina-spinel brick: (**a**) fracture surface, (**b**) polished cross-section (point 1: alumina, and point 2: spinel).

SEM micrograph of alumina-spinel brick in fracture and cross-section views are presented in Figure 8a,b, respectively. EDS measurements conducted at polished cross-sections were employed to obtain elemental analysis on a definite grain. As indicated in Table 5, point 1 represents the area of the alumina grain, while the composition in point 2 represents the non-stoichiometric Al-rich spinel distributed within the matrix, characterized by Al/Mg of 3.3 (Al/Mg is 2 in MgAl_2_O_4_), which confirms the XRD results.

### 3.4. Alumina-Spinel Refractory after Corrosion Test

**Figure 9:**
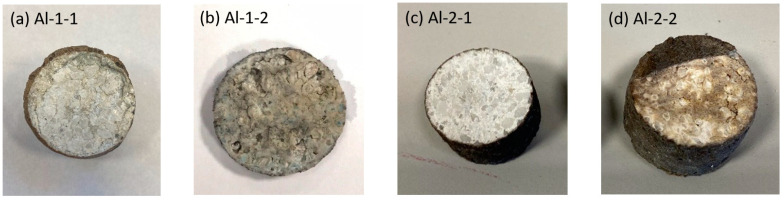
The alumina-spinel refractory after corrosion by slag R (**a**) Al-1-1 (1350 °C) and (**b**) Al-2-1 (1450 °C), and slag S (**c**) Al-2-1 (1350 °C) and (**d**) Al-2-2 (1450 °C).

Figure 9a–d show macro-photographs of all the samples after corrosion testing at 1350 °C and 1450 °C for 10 h. For corrosion at a lower temperature, both slags remained on the samples and no significant changes in the sample dimensions were registered. Even after cutting, the internal parts of the sample Al-1-1 (corroded by R slag) and Al-2-1 (corroded by S slag) remained unaltered, which shows minor infiltration of slag towards the refractory at 1350 °C. 

However, at an increased temperature of 1450 °C a low amount of slags remained on the external parts of both samples Al-1-2 and Al-2-2 due to intense infiltration of slag to refractory, and the sample expansion registered high at 25%. 

#### 3.4.1. X-ray Diffractometry

Due to the minor infiltration of slag into samples at 1350 °C, two different areas in the samples were selected for XRD examination, namely the edge (external) area and core (internal) area. The first sample was collected from the interaction zone where the slag was in direct contact with the surface of the refractory, while the second sample was gathered from the interior area, intact by slag as observed with naked-eye observation. Thanks to this separation, the difference in phases between the interaction zone and the un-reacted zone can be distinguished and investigated. 

**Figure 10:**
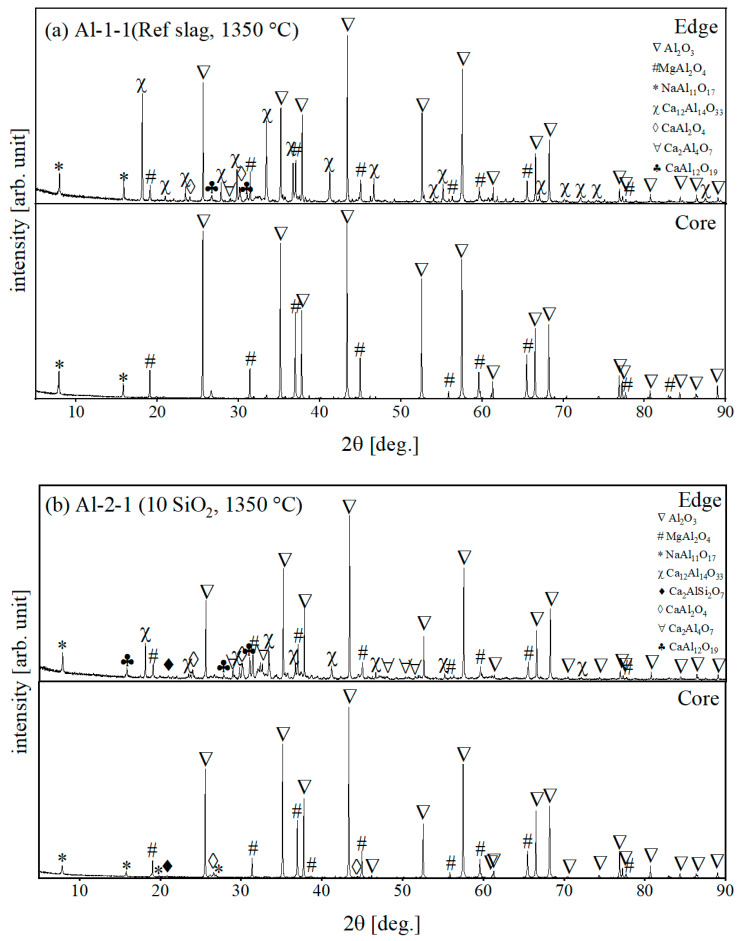
XRD patterns of (**a**) Al-1-1 (slag R) and (**b**) Al-2-1 samples (slag S), for 1350 °C.

XRD patterns of corroded samples taken from the edge and core parts are presented in Figure 10a,b for R and S samples, respectively. For corrosion at 1350 °C by S slag, gehlenite (Ca_2_Al_2_SiO_7_, C_2_AS) was identified in the core and edge parts of sample Al-2-1, confirmed by the presence of a characteristic reflex at 22° of 2θ. This is attributed to the increased content of SiO_2_ in the composition of slag S, which reacted with components of refractory, causing its corrosion. Based on the SiO_2_-CaO-Al_2_O_3_ ternary phase diagram [16,17], gehlenite is the low melting phase (T_m_ = 1593 °C) located in the eutectic zone, and it can generate the liquid phase during the high-temperature corrosion process. Apart from C_2_AS in Al-2-1, phase composition in the core parts of both samples was similar. 

In the edge parts of both samples, the original components of refractory (αAl_2_O_3_, spinel, βAl_2_O_3_) and slag (C_12_Al_14_O_33_) were detected. Additionally, new aluminates appeared in low amounts in the edge parts of both samples; namely: CaAl_2_O_4_ and CaAl_12_O_19_. Mayenite from slag was not detected in the core parts of either sample. 

**Figure 11:**
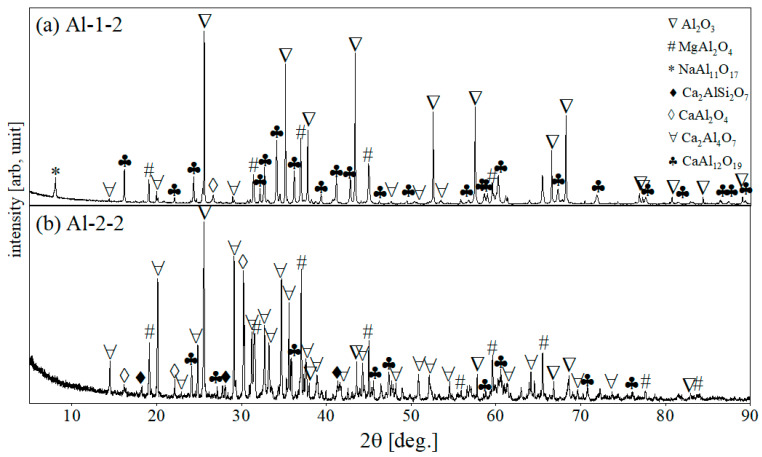
XRD patterns of (**a**) Al-1-2 (slag R) and (**b**) Al-2-2 samples (slag S), for 1450 °C.

The XRD patterns of Al-1-2 and Al-2-2 samples after corrosion at a higher temperature of 1450 °C are depicted in Figure 11a,b. After corrosion at 1450 °C, the core and edge parts of the sample were equally corroded by visual assessment; thus, only one averaged sample was subjected to XRD analysis. As observed from XRD patterns, there is an evident difference in phase composition between samples Al-1-2 (R slag) and Al-2-2 (S slag). Specifically, different types of new aluminate products appeared as a result of corrosion. 

In both samples, calcium monoaluminate (CA), calcium dialuminate (CA_2_), and calcium hexaluminate (CA_6_) were formed. However, peaks of gehlenite (C_2_AS) were observed in the XRD pattern of only Al-2-2, which results from the increased silica content (10 wt.% of SiO_2_) in slag S. 

Another difference in the XRD patterns between samples Al-1-2 and Al-2-2 is the intensity of the peaks for CA, CA_2_, and CA_6_ phases. The XRD pattern of sample Al-1-2 (less SiO_2_ content) contains higher peaks for CA and CA_6_ phases, while the pattern of Al-2-2 is characterized by more peaks of CA_2_. As can be seen by the predominant reflexes, a significant amount of CA_6_ was formed in Al-1-2 (R), while in Al-2-2 the main corrosion product was CA_2_. CA was identified in both samples in low amounts. From this, it can be seen that the greater formation of new aluminates at 1450 °C, compared to 1350 °C, is observed due to enhanced diffusion of Ca^2+^ towards components of Al_2_O_3_-MgAl_2_O_4_ refractory. The appearance of new phases was followed by decreasing the intensity of reflexes of the original refractory phases, due to their corrosion. No reflexes in after-corrosion were found for mayenite, the main phase of corrosive slag, confirming its total reaction. 

In contrast to composition in the edge of samples A-1-1 and Al-2-1, in the XRD pattern of the samples, in Al-1-2 and Al-2-2 the peaks of calcium aluminate (CA) were considerably reduced while the peaks of calcium dialuminate (CA_2_) and calcium hexaluminate (CA_6_) raised up, which indicates progressing diffusion at 1450 °C of calcium ions (Ca^2+^) to alumina (Al_2_O_3_), forming Al_2_O_3_-richer calcium aluminates. In addition, the formation of calcium hexaluminate (CA_6_) reveals that the corrosion proceeds in the direction of the equilibrium conditions, as the excess of alumina is still present in the system. This behavior was correlated with decreasing numbers as well as the lower intensity of reflexes for αAl_2_O_3_, which is related to the dissolution of alumina grains in the molten slag during the corrosion process.

#### 3.4.2. SEM/EDS Observations

**Figure 12:**
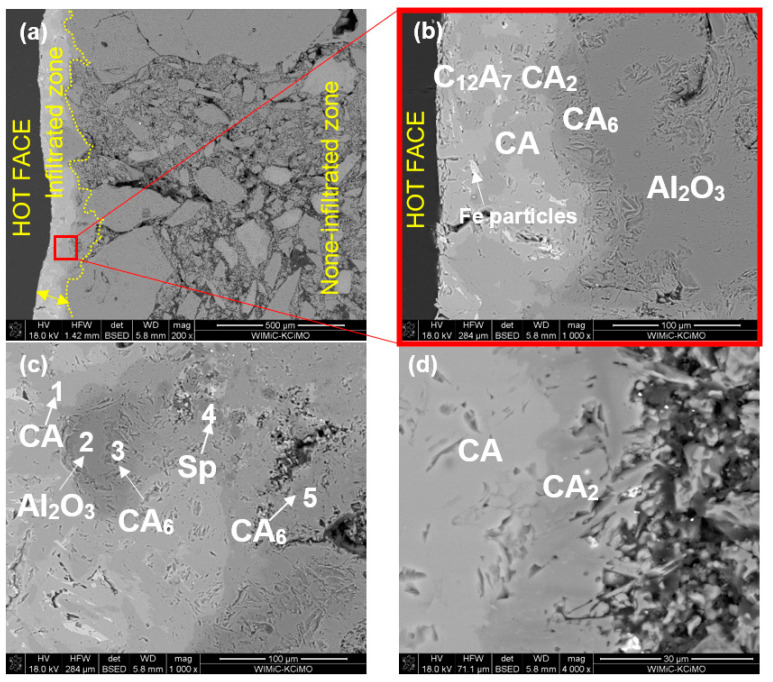
SEM images of sample Al-1-1 corroded by slag R (1350 °C, 10 h); (**a**,**b**): hot-face (slag/refractory interface); (**c**,**d**) higher magnification of interface zone.

Figure 12a–d show the SEM images of sample Al-1-1 corroded at 1350 °C. The phases indicated in the images were determined based on the chemical composition measured by EDS at a specific micro-area shown in Table 6. In this approach, we discovered secondary phases in the reaction zone produced due to corrosion of refractory by metallurgical slag. 

As can be observed from the image of sample Al-1-1 corroded by R slag at 1350 °C (Figure 12a–d), slag infiltration affected the matrix compactness. The contact layer—mostly infiltrated by the slag—constituted about 150 µm. The magnified image (Figure 12b) clearly shows that this layer is composed of various types of calcium aluminates, located in the following direction from the contact zone: C_12_A_7_ (original slag component), CA, CA_2_, CA_6_. This confirms the progressive reaction of calcium aluminate from slag with alumina from refractory (phases in phase diagram Al_2_O_3_-CaO in the order of appearance: A-C_3_A-C_12_A_7_-CA-CA_2_-CA_6_-C). In the depth above 150 µm from the contact zone, only Al_2_O_3_ was observed (Figure 12b). CA_6_ crystallized in the form of flake crystals (Figure 12b,c). Such platelet morphology of CA_6_ has previously been observed by numerous researchers [12,13,18,19]. The number of new phases was not significant in the volume of material.

**Figure 13:**
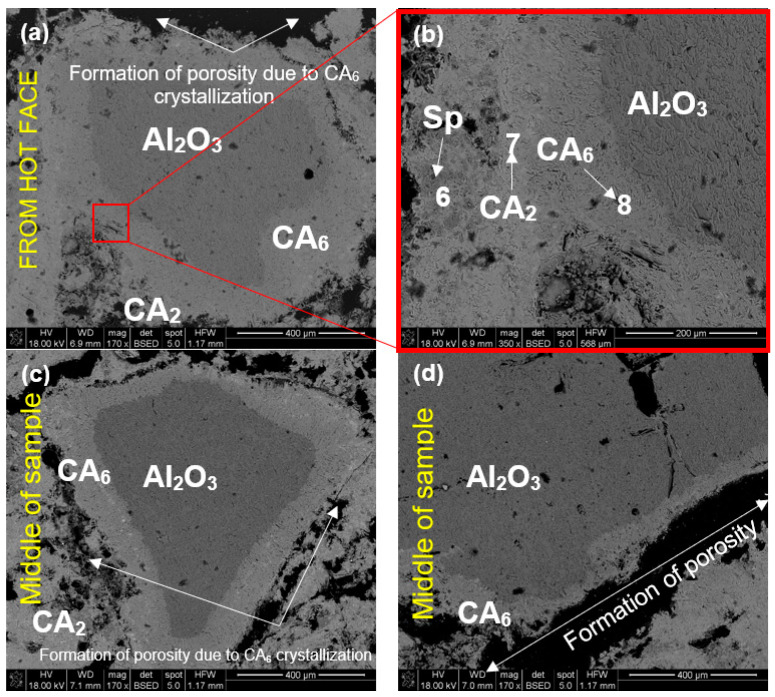
SEM images of sample Al-1-2 corroded by R slag (1450 °C, 10 h); (**a**) hot-face zone, (**b**) higher magnification of hot-face zone, (**c**,**d**) middle of the sample (core zone).

The same sample but corroded at a higher temperature of 1450 °C (Al-1-2) was much more porous when compared to samples corroded at 1350 °C, as can be seen in Figure 13. This porosity is caused by the massive formation of platelet CA_6_ crystals, which formed especially around Al_2_O_3_ grains, resulting in their separation from other components of the microstructure (Figure 13a,c,d). Spinel grains were dispersed among CA_2_ polycrystals (Figure 13b). The progressive diffusion of Ca^2+^ from slag toward refractory, and Al^3+^ from refractory toward slag, resulted in the formation of calcium aluminates with increased Al content, which are evident from the generated layered microstructure zone CA_2_/CA_6_/Al_2_O_3_ in Figure 13b. 

**Figure 14:**
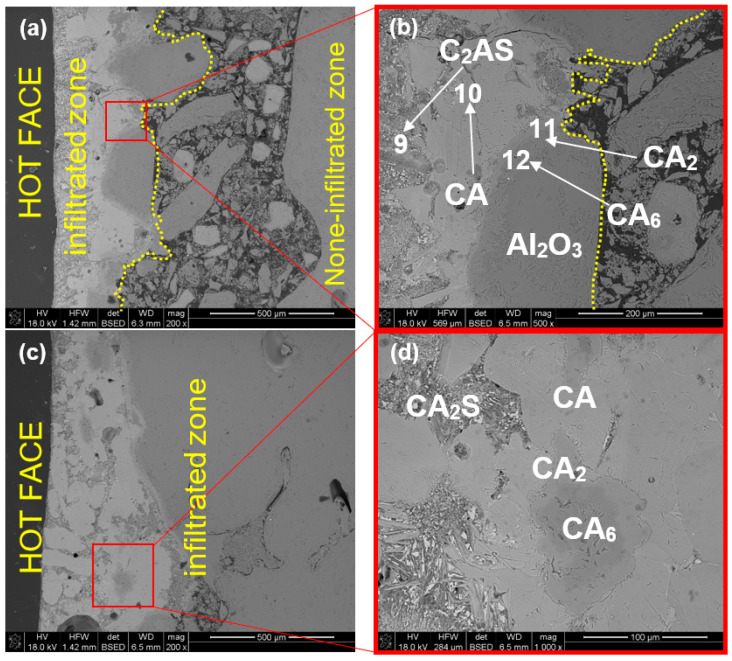
SEM images of sample Al-2-1 corroded by slag S (1350 °C, 10 h); (**a**,**c**) hot-face zone of the impregnated area, (**b**,**d**) high magnification of ho-face zone (slag/refractory interface).

Figure 14a–d illustrate the SEM images of the Al-2-1 sample corroded by slag S at 1350 °C for 10 h. In this case, the infiltrated zone was much broader, from about 270 to over 700 µm. Gehlenite, C_2_AS, was additionally detected in the after-corrosion refractory, which occurred prior to calcium aluminate layers CA, CA_2,_ CA_6_ (Figure 14b,d). C_2_AS, not observed for R slag, formed from the side of liquid slag S. This confirms the XRD results, which showed a small amount of gehlenite (Figure 10b). The calcium monoaluminate, CA, was observed in a gap between gehlenite, C_2_AS, and calcium dialuminate, CA_2_, in Figure 14b. Calcium hexaluminate, CA_6_, and calcium dialuminate, CA_2_, surrounded tightly the alumina aggregate as a result of intensive Ca^2+^ diffusion towards refractory grains. All the results were confirmed by the EDS analysis shown in Table 6.

**Figure 15:**
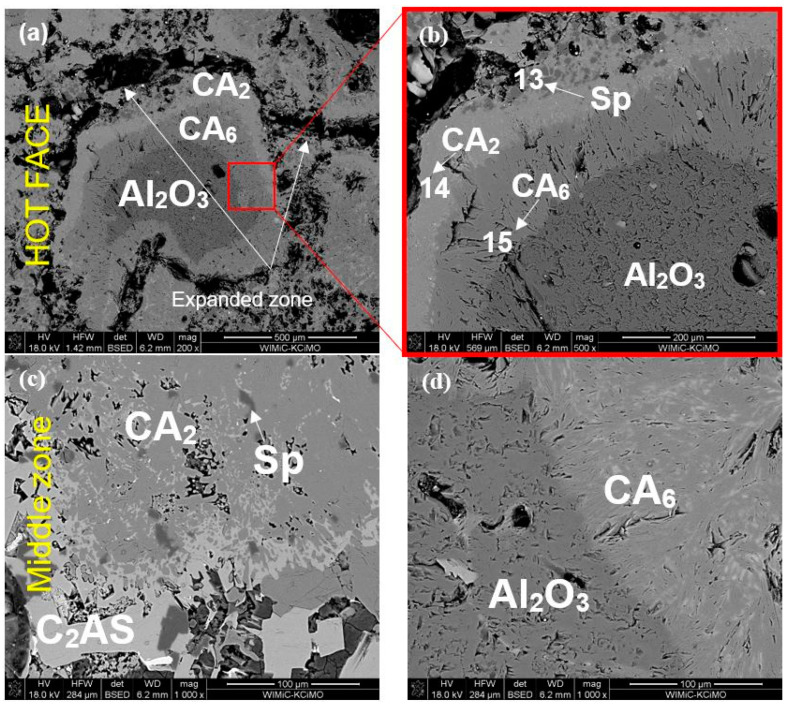
SEM images of sample Al-2-2 corroded by slag S (1450 °C, 10 h); (**a**) hot-face zone of the Al-2-2 sample, (**b**) higher-magnification of ho-face zone, (**c**) middle of the Al-2-2 sample, (**d**) higher magnification of middle zone.

After corrosion testing at 1450 °C (Al-2-2, Figure 15a–d), more progressive corrosion is clearly visible in the SEM images. A greater rate of diffusion is confirmed by the clearly distinguishable rings of secondary aluminates around alumina grains (Figure 15b). Intensive crystallization of platelet morphology CA_6_ left great amounts of porosity. C_2_AS was still present at 1450 °C (Figure 15c).

## 4. Discussion

### 4.1. Slag Viscosity

For evaluating the slag viscosity, different methods and models have been used in the literature. One of the most common models is the slag basicity, defined as a weight ratio of basic to acid oxides, according to Equation (2) [8].

(2) $$\%B=\frac{\mathrm{wt}.\%\mathrm{CaO}+\mathrm{wt}.\%\mathrm{MgO}+\mathrm{wt}.\%\mathrm{MnO}+\mathrm{wt}.\%\mathrm{FeO}\;\left(\mathrm{Basic}\;\mathrm{oxides}\right)}{\mathrm{wt}.{\%\mathrm{SiO}}_{2}+\mathrm{wt}.{\%\mathrm{Al}}_{2}O_{3}+\mathrm{wt}.{\%\mathrm{Fe}}_{2}O_{3}+\mathrm{wt}.{\%P}_{2}O_{5}\;\left(\mathrm{Acid}\;\mathrm{oxides}\right)}$$

The XRF results presented in Table 1 indicate that the basicity of R and S slags are 0.99 and 0.88, respectively. Hence, slag S should possess higher viscosity and lower infiltration tendency compared to slag R [8]. Therefore, the increased viscosity of silica-enriched slag S was the reason for its higher flow point when compared to R slag in high-temperature microscopy testing (S-1345 °C and R-1320 °C).

According to the theory of Herasymenko [20], the slag structure can be assumed to be an ionic structure in the liquid state with cations including atoms of Si, P, Fe, Ca, Fe, Mn, Mg, Na, and non-metallic anions of O, S, and F. The small ions, with high charge, can attract oxygen to form complex structures, e.g., SiO_4_, PO_4_, AlO_3_, FeO_2_, and Fe_2_O_5_ in stable tetrahedral arrangements. These acid structural groups share the oxygen (so-called oxygen bridge) and are considered network formers; thus, they are responsible for the increased slag viscosity. Conversely, basic oxides such as CaO, MgO, FeO, and MnO release O^2−^ to the slag, thus breaking the network. 

**Figure 16:**
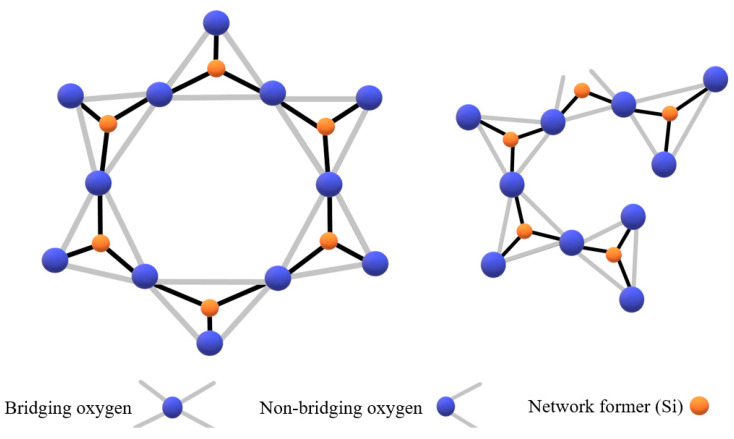
Network of Si-O tetrahedral in (**a**) crystalline and (**b**) molten slag [21].

SiO_2_ is a common component of numerous metallurgical slags. The structure of the liquid slag can be explained with respect to the silica addition. SiO_2_ is an acid oxide and network former in the structure of slag. Furthermore, each Si^4+^ is surrounded tetrahedrally by O^2−^ ions, and each O^2−^ ion is connected to the other two oxygen ions. Because of the possibility of bonding in four tetrahedral directions, the slag structure exists as a three-dimensional polymerized network. Based on that, the viscosity of S slag is considered higher than R slag. Figure 16 shows the Si-O tetrahedral network in crystalline and molten slag.

**Figure 17:**
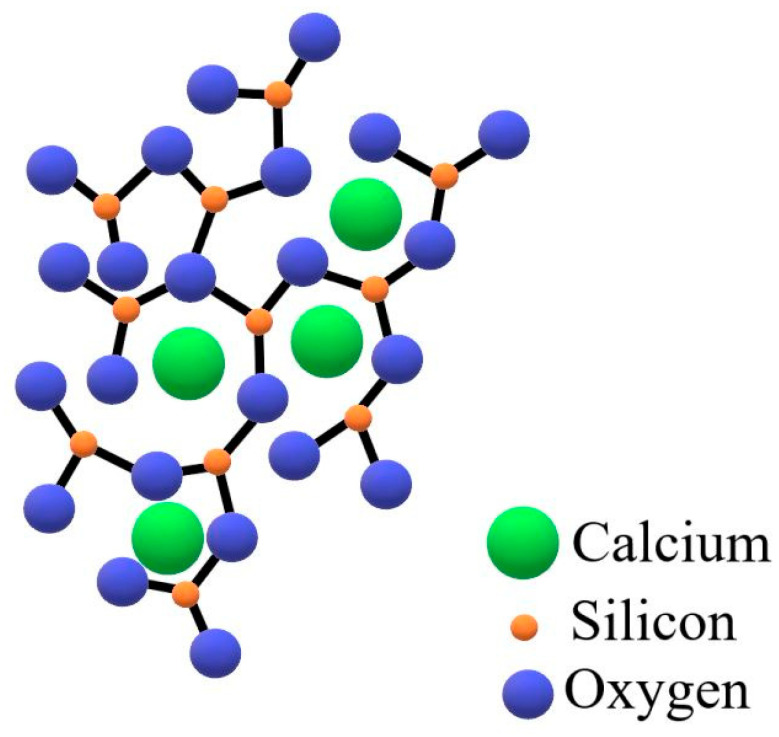
Structure of CaO-SiO_2_ liquid slag [21].

The addition of basic oxides such as CaO (network breakers) causes revealing of the free oxygen O^2−^ and Ca^2+^ cations to a slag, which breaks Si-O bonds. Non-bridging oxygens de-polymerize the liquid slag and, consequently, make a charge balance for the CaO-SiO_2_ system. If the slag becomes totally de-polymerized, all tetrahedral connections break down. Then, the slag is composed of free SiO_4_^4−^, O^2−^, and Ca^2+^. Figure 17 illustrates the structure of CaO-SiO_2_-based slag, with tetrahedral connections broken in the system. 

### 4.2. Slag-Refractory Interactions

**Figure 18:**
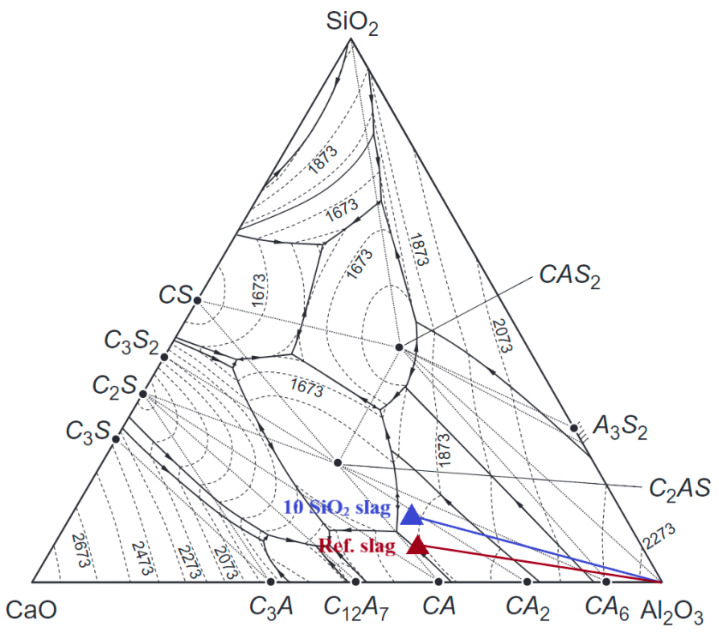
Ternary phase diagram of CaO-Al_2_O_3_-SiO_2_ [25]. The starting compositions of the slag R (Ref. slag) and slag S (10SiO_2_) are indicated in the diagram.

Based on the SEM images of the corroded samples Al-1-1 and Al-1-2 (Figure 12 and Figure 13), calcium aluminate layers are distributed around the tabular alumina grains, which indicates the indirect dissolution mechanism of alumina-spinel refractory by metallurgical slag. The indirect dissolution of refractories has been studied by numerous authors [18,22,23,24]. According to the ternary phase diagram for CaO-Al_2_O_3_-SiO_2_ (Figure 18) and XRD results, alumina aggregates in this work were indirectly dissolved forming CaAl_2_O_4_ (CA), CaAl_4_O_7_ (CA_2_), and CaAl_12_O_19_ (CA_6_). CA_6_/CA_2_ interfaces were clearly identified at the edges of tabular alumina grains by SEM/EDS. Previous studies on the corrosion of high-alumina spinel refractories [15,22] indicated the formation of dense layers around the coarse alumina grains. These layers consisted of CA, CA_2_, and CA_6_, and were suggested to impede corrosion during the wear process. 

**Figure 19:**
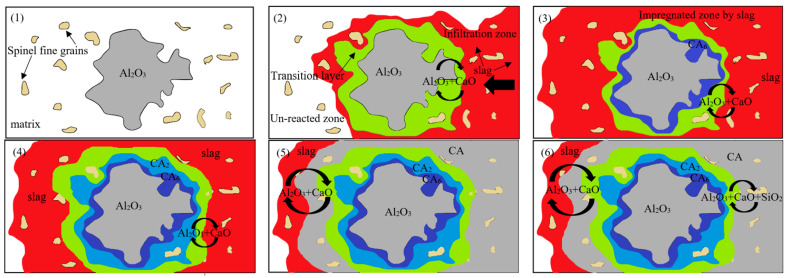
Model of corrosion mechanism of alumina-spinel refractory by metallurgical slag (green—transition layer in each corrosion stage, red—slag); (1) un-infiltrated Al-Sp microstructure, (2) first contact between CaO-Al_2_O_3_-SiO_2_ (CAS) slag and Al-Sp refractory, (3) formation of CA_6_ layer around alumina aggregates, (4) formation of CA_6_, CA_2_ layers (5) formation of CA_6_, CA_2_, and CA layers according to precipitation-dissolution mechanism.

According to Khajornboon et al. [17], CA (sintered at 800–1000 °C) and CA_2_ (sintered at 1000–1300 °C) phases are incompatible at 1450 °C, and from a thermodynamic point of view, they react with Al_2_O_3_ aggregate present in the microstructure forming CA_6_. Moreover, due to phenomena occurring at interface CA_6_/CA_2_—diffusion of Ca^2+^ from CA_6_ and the dissolution of Al_2_O_3_—CA_6_ thickness grows. In addition, because of the diffusion of Ca^2+^ from slag to the interface slag/CA_2_, the CA_2_ layer grows. This phenomenon of indirect corrosion of alumina-spinel refractory is presented by model in Figure 19. 

The results obtained in this work show that CA_6_ previously formed around Al_2_O_3_ grains acts as an obstacle for further reaction. As can be observed in the SEM micrographs, the alumina aggregates are progressively attached by the calcium hexaluminate layer, and because of CA_6_ formation, the erosion of alumina grains reduces, thus improving their corrosion resistance.

According to the results shown in this study for Al_2_O_3_-MgAl_2_O_4_ refractory, and confirmed by the literature [8,23,24], CA_6_ and CA_2_ layer formation can prevent further corrosion of refractory aggregate. Nevertheless, it should be noted that there is about a 3.1% difference in density between CA_6_ (3.78 g/cm^3^) and alumina (3.95 g/cm^3^), which contributes to the volumetric expansion during corrosion [6,24,26]. Consequently, CA_6_ can cause the initiation of cracks, which intensifies the slag infiltration for longer corrosion periods. Therefore, despite the physical properties of alumina-spinel brick (porosity and density), a higher quantity of CA_6_—formed during corrosion—can facilitate the slag penetration followed by deterioration of the functional properties of the refractory [16]. 

According to De Bilbao et al. [22,23], CA_2_ phase is not a stable phase at elevated temperatures. Based on the dissolution/precipitation mechanism, the melted slag forms CA_6_ and CA_2_ phases in the initial stage of corrosion. In the first stage of corrosion, solid/solid reactions proceed, and since the slag contains Ca^2+^ and O^2−^ ions, which diffuse and react at interfaces Al_2_O_3_/CA_6_ and CA_6_/CA_2_, the alumina layer is gradually dissolved, as shown in Figure 19. Moreover, the external CA_2_ layer dissolves in the slag according to Equation (3).

(3) $$CA_{2}\to C_{\left(l\right)}+2A_{\left(l\right)}$$

The liquid alumina Al_(l)_, formed according to Equation (3), is therefore indirectly dissolved and revealed to slag. Then, Ca^2+^ cations are transported from the CA_2_/slag interface toward the alumina grains and enter the CA_2_/CA_6_ interface, which is followed by reactions according to Equations (4) and (5):

(4) $$3{\mathrm{CA}}_{2}\to 2C+{\mathrm{CA}}_{6}$$

(5) $${\mathrm{CA}}_{6}+2C\to 3{\mathrm{CA}}_{2}$$

Reaction proceeding according to Equation (4) shifts the interface toward refractory alumina grain, while reaction proceeding according to Equation (5) follows in the opposite direction. Finally, the alumina aggregate shrinks, which facilitates cation diffusion through the CA_6_ layer and enhances the reaction at the internal interface Al_2_O_3_/CA_6_, as shown by Equation (6).

(6) $$6A+C\to {\mathrm{CA}}_{6}$$

The crystal structures of CA_2_ and CA_6_, forming around alumina aggregates, are monoclinic and hexagonal, respectively. Therefore, the highest density of oxygen packing belongs to CA_6_ and thanks to the crystallized close-packed system its cation diffusivity is lower compared to CA_2_. Consequently, the content of cations that can leave the CA_2_/CA_6_ interface is lower than the cations that arrive in the CA_2_/CA_6_ interface. Therefore, the CA_2_/CA_6_ interface shifts toward the alumina aggregate, which is related to the increasing thickness of the CA_2_ mono-layer. Then, the CA_2_ mono-layer dissolves in the slag and its growth is limited as well. 

The corrosion mechanism is shown in Figure 19. The green layer indicates the transitional layer in each step of corrosion. According to image (1), before the slag impregnation the alumina coarse grain (grey) is attached by the fine alumina-spinel grains (yellow) forming the matrix. At the initial corrosion stage (2), when the slag (red) infiltration begins, Al_2_O_3_ (from refractory) reacts with CaO (from slag) and, as a result, a transition layer (green) starts to form around the alumina grain. Subsequently, the impregnated zone extends and the calcium hexaluminate layer (CA_6_, purple) forms around alumina aggregate as shown by (3). By prolonging the time of corrosion, calcium dialuminate (CA_2_, blue) and calcium monoaluminate (CA, light grey) layers form around the A aggregate and, through this, the alumina aggregate is preserved from further corrosion. Finally, via extending the transitional zone, the chemical reactions continue until reaching thermodynamic equilibrium conditions. 

The Rietveld method was employed to determine the quantity of each calcium aluminate phase in all samples (Al-1-1, Al-1-2 and Al-2-1, Al-2-2); the results are shown in Table 7. The amounts of gehlenite, C_2_AS, in samples corroded by slag S (Al-1-2 and Al-2-2) were at a comparable levels of 11.5% and 10.6% for 1350 °C and 1450 °C, respectively. In contrast, about two times lower amounts of gehlenite formed in refractory corroded by slag R, which results from two times lower SiO_2_ content in the original slag (5% SiO_2_).

Gehlenite was formed as a corrosion product of a reaction between SiO_2_ and CaO from slag with Al_2_O_3_ from refractory. C_2_AS is a phase located in the ternary phase diagram SiO_2_-CaO-Al_2_O_3_ (Figure 18), which melts at 1593 °C, thus increasing the content of the liquid in the matrix of material operating at high temperatures in steel devices. 

The raised temperature of heat treatment causes the greater formation of CA_6_, for both R slag and S slag. For 1350 °C—regardless of the type of slag—the corroded refractory contained similar content of CA_6_ of about 24%. However, for corrosion at 1450 °C, the amount of CA_6_ significantly increased (to 37% for R and to 50% for S) due to a greater rate of diffusion at higher temperatures. This will influence the compactness of the refractory during its operation.

## 5. Conclusions

This study showed the corrosion mechanism of alumina-spinel refractory by secondary metallurgical slag (B = 0.99) and slag with 10 wt.% addition of SiO_2_ content (B = 0.88). For this purpose, an alumina-spinel disc sample was coated with a shell of slag to directly contact the external faces of the refractory sample. Subsequently, the coated samples were subjected to corrosion at 1350 and 1450 °C in an oxidizing atmosphere. The after-corrosion materials were analyzed in view of the alterations in their structure and microstructure. The following conclusions can be drawn from the results:

- The corrosion mechanism of alumina-spinel material tested against acid metallurgical slags is based on the indirect dissolution of alumina grains constituting the principal component of the refractory.
- The indirect dissolution of alumina covers the formation of new calcium aluminate layers around alumina grains, including CA_2_ and CA_6_, for both corrosive slags and heating at both 1350 °C and 1450 °C. By increasing the temperature to 1450 °C, the infiltration of refractory by slag increases; specifically, Ca^2+^ diffuses more intensively towards refractory followed by the formation of CA_2_ and CA_6_.
- CA_6_ forms in platelet morphology and by expansion causes loosening of the microstructure, especially at increased temperatures when its amount is doubled.
- The low melting phase of gehlenite, Ca_2_Al_2_SiO_7_, forms in phase composition of alumina-spinel samples corroded by both slags, and its amount is doubled for silica enriched S slag containing 10% SiO_2_.

## Figures and Tables

**Table 1:** XRF chemical composition of slags used in the study.

Component (wt.%)	R Slag	S Slag
CaO	48.5	45.6
Al_2_O_3_	40.9	38.4
SiO_2_	4.20	10.0
Fe_2_O_3_	2.20	2.10
TiO_2_	1.90	1.80
MgO	0.60	0.60
**P_2_O_5_**	0.47	0.42
**SO_3_**	0.40	0.38
**K_2_O**	0.33	0.29
**Cr_2_O_3_**	0.05	0.05
**MnO**	0.08	0.05
**SrO**	0.05	0.04
**ZrO_2_**	0.09	0.08
**Na_2_O**	0.21	0.18
**Cl**	0.02	0.01

**Table 2:** Characteristics of alumina-spinel.

Element	Chemical Composition by XRF (wt.%)
Al_2_O_3_	87.7
MgO	5.8
SiO_2_	4.8
Na_2_O	0.5
Fe_2_O_3_	0.3
CaO	0.2
Others	0.8
Bulk density (g/cm^3^)	3.08
Apparent porosity (%)	18.42

**Table 3:** Program of corrosion testing.

Sample Name	Slag Type	Temperature (°C)	Dwell Time (h)	Atmosphere
Al-1-1	R	1350	10	Oxidizing (air)
Al-1-2	R	1450	10	
Al-2-1	S	1350	10	
Al-2-2	S	1450	10	

**Table 4:** EDS analysis of micro-areas indicated in Figure 5.

Point	Chemical Composition by EDS, at.% *						Ratio Ca/Al **	Phase
	Ca	Al	Si	Mg	Ti	Fe		
1	21.6	38.4	0.3	0.1	-	-	0.6	C_12_A_7_
2	22.6	35.4	2.3	0.3	0.9	-	0.6	C_12_A_7_
3	25.1	22.9	7.2	0.8	2.8	1.5	1.1	C_3_A-C_12_A_7_

* the rest to 100% is oxygen; ** theoretical Ca/Al ratio in aluminates: C_3_A-1.5, C_12_A_7_-0.9, CA-0.5, CA_2_-0.3, CA_6_-0.1.

**Table 5:** EDS analysis of micro-areas indicated in Figure 8.

Point	Element (at.%) *	
	Mg	Al
1	-	53
2	13.8	45.5

* the rest to 100% is oxygen.

**Table 6:** EDS chemical composition in selected micro-areas indicated in Figure 12, Figure 13, Figure 14 and Figure 15.

Point	Elemental Composition (at.%) *					Assigned Phase
	Mg	Al	Si	Ca	Ti	
1	0.27	38.36	-	23.11	-	CA
2	-	52	-	-	-	Al_2_O_3_
3	1.05	50.04	-	4.38	-	CA_6_
4	17.11	42.05	-	0.37	-	Sp
5	2.38	51.38	-	5.96	-	CA_6_
6	16.27	41.65	-	0.29	-	Sp
7	0.93	43.99	45	13.17	-	CA_2_
8	1.03	46.45	0.56	4.37	-	CA_6_
9	1.43	15.24	5.95	13.30	3.03	C_2_AS
10	0.70	34.48	0.44	14.40	-	CA
11	-	37.18	-	8.23	-	CA_2_
12	-	41.83	-	2.99	-	CA_6_
13	15.69	40.88	-	-	-	MA
14	-	41.18	-	13.87	-	CA_2_
15	-	49.27	-	5.09	-	CA_6_

* The rest to 100% is oxygen.

**Table 7:** Rietveld analysis of the test slag.

Slag	1350 °C			1450 °C		
	CA_6_	CA_2_	C_2_AS	CA_6_	CA_2_	C_2_AS
R slag	23.6	40.6	6.2	36.5	40.9	6.1
S slag	24.6	38.5	11.5	49.5	25.3	10.6

## Data Availability

Not applicable.
